# Overexpression of high mobility group box 1 and 2 is associated with the progression and angiogenesis of human bladder carcinoma

**DOI:** 10.3892/ol.2012.1091

**Published:** 2012-12-27

**Authors:** WEI WANG, HAOWEN JIANG, HECHEN ZHU, HU ZHANG, JIAN GONG, LIMIN ZHANG, QIANG DING

**Affiliations:** 1 Institute of Urology, Huashan Hospital, Fudan University, Shanghai 200040, P.R. China; 2 Departments of Urology, Huashan Hospital, Fudan University, Shanghai 200040, P.R. China; 3 ICU, Huashan Hospital, Fudan University, Shanghai 200040, P.R. China

**Keywords:** high mobility group box, bladder carcinoma, angiogenesis

## Abstract

High mobility group box 1 (HMGB1) and HMGB2 overexpression has been observed in several human tumor types, and is involved in cancer progression and prognosis. However, the clinicopathological significance of HMGB1 and HMGB2 expression in bladder carcinoma (BCa), particularly the involvement of these proteins in angiogenesis, remains unclear. In the present study, immunohistochemistry and real-time polymerase chain reaction (PCR) of HMGB1 and HMGB2 in 64 BCa patients revealed that HMGB1 and HMGB2 were overexpressed in BCa tissues compared with normal tissues, and were correlated with tumor clinical stage and pathological grade. In addition, correlation analysis of vascular endothelial growth factor (VEGF) and microvessel density (MVD) counts indicated that the overexpression of HMGB1 and HMGB2 was also correlated with angiogenesis. We conclude that HMGB proteins act as key regulators in the progression and angiogenesis of bladder carcinoma, and serve as potential diagnostic and therapeutic targets.

## Introduction

Bladder carcinoma (BCa) is the most common genitourinary malignancy worldwide. The disease has been annually diagnosed in 55,600 males and 17,910 females in the USA and is the fourth most common type of cancer in males and the eighth most common in females (1). Fifty to seventy percent of initially diagnosed BCa patients experience a recurrence within 5 years; however, 10% progress to invasive BCa (2). The diverse biological behavior of BCa has forced current guidelines to recommend intense follow-up and invasive treatment, as no reliable method to determine the recurrence and invasive potential of such tumors has been identified (3). It is important to predict the invasive behavior of BCa in clinical studies, and specific molecular markers that may serve as credible potential prognostic factors are required.

High mobility group box (HMGB) proteins are ubiquitous, abundant nuclear proteins with diverse functions in the cell. The proteins were first purified from nuclei in the 1970s and termed ‘high mobility group’ (HMG) proteins to reflect their rapid mobility in sodium dodecyl sulfate-polyacrylamide gel electrophoresis (SDS-PAGE) gels (4,5). HMGB1 and HMGB2 are the main members of the HMGB protein family. Unlike HMGB1, HMGB2 is highly expressed during embryogenesis, but has limited expression in adult organs, mainly in the lymphoid organs and testes. HMGB1 and HMGB2 are highly conserved with >80% amino acid identity and similar molecular structures; both proteins have a tripartite domain organization, consisting of two DNA-binding domains, the HMG-boxes A and B, and acidic C-terminal tails of variable length (6,7). HMGB1 and HMGB2 have indistinguishable biological properties within the nucleus, such as binding to DNA without sequence specificity and regulating transcription, replication, DNA repair and recombination (8–10). HMGB1 and HMGB2 are important in growth and development. An early study demonstrated that HMGB1 knockout mice did not survive within 24 h after birth, due to hypoglycemia, and they exhibited a defect in the transcriptional function of the glucocorticoid receptor. Additionally, the HMGB2 knockout mice survived into adulthood, although spermatogenesis was impaired (6).

In cancer chemotherapy, HMGB1 and HMGB2 may act as sensors of DNA modification and facilitate p53 phospho rylation following exposure to genotoxic stress, thus becoming newly identified components of the DNA damage signaling cascade and providing novel promising targets for chemotherapeutic intervention (7,11). HMGB1 has also been demonstrated to be released into the extra cellular medium and to exhibit important effects in the mediation of tumor growth, angiogenesis and metastasis (12,13). Overexpression of HMGB1 and HMGB2 has been observed in several human cancer types, including hepatocellular (14), skin squamous cell (15), prostate (16), gastrointestinal (12,17) and breast carcinomas (18,19). HMGB1 has been identified to be a latent pro-angiogenic factor in cancer progression and angiogenesis; it has been observed to initiate the production of angiogenic factors, such as vascular endothelial growth factor (VEGF) (20,21). As a surrogate marker for angiogenesis, microvessel density (MVD) has been demonstrated to be predictive of progression and a poor prognosis of BCa (22,23). Although there has been extensive characterization of the various roles of HMGB1 in cancer, considerably less is known regarding the involvement of HMGB2 in carcinogenesis, including its angiogenic effects and precise signaling pathways.

The present study investigated the clinicopathological significance of HMGB1 and HMGB2 expression in human BCa by quantitative real-time polymerase chain reaction (PCR) and immunohistochemistry. Furthermore, the correlations between the expression of HMGB1/HMGB2 and VEGF as well as MVD counts have been analyzed, to explore the angiogenic role of HMGB1 and HMGB2 in BCa.

## Materials and methods

### Patients and tissue samples

Tumor tissue samples were collected from 64 patients with BCa and 15 normal bladder tissue samples were obtained from the Department of Urology, Huashan Hospital between January 2010 and July 2011. All tissues were confirmed by histological examination of sequential sections. Tumor staging was determined according to the sixth edition of the tumor node metastasis (TNM) classification of the International Union Against Cancer. Clinical information regarding the samples is described in detail in Table I. Ethical approval was obtained from the research ethics committee of Huashan Hospital, and written informed consent was obtained from all patients.

### Real-time PCR

Total RNA was isolated from the tumor and normal tissues using TRIzol reagent (Invitrogen Life Technologies; Carlsbad, CA, USA). Total RNA (2 *μ*g) was reverse transcribed to cDNA using M-MLV reverse transcriptase (Promega Corp., Madison, WI, USA). A typical 25 *μ*l reaction mixture contained 12.5 *μ*l 2X One Step SYBR RT-PCR buffer (Takara Bio. Inc., Shiga, Japan), 11 *μ*l of water, 0.5 *μ*l of template and 1 *μ*l of specific primers. The primer sequences were as follows: forward: 5′-ATATGGCAAAAGCGGA CAAG-3′ and reverse: 5′-GCAACATCACCAATGGACAG-3′ for HMGB1; forward: 5′-CGTTCCTCCCAAAGGTGATA-3′ and reverse: 5′-TCTTTGGCTGACTGCTCAGA-3′ for HMGB2; forward: 5′-GGCGGCACCACCATGTACCCT-3′ and reverse: 5′-AGGGGCCGGACTCGTCATACT-3′ for β-actin. An initial denaturation/activation step (15 sec at 95°C) was followed by 40 cycles (5 sec at 95°C and 30 sec at 60°C). The relative expression of HMGB1 and HMGB2 mRNA was calculated with the 2^−ΔΔCt^ method and normalized using the β-actin mRNA expression level. All experiments were performed in triplicate.

### Immunohistochemistry and evaluation

Formalin-fixed and paraffin-embedded tissue sections (5 mm) were dewaxed with xylene and rehydrated through an ethanol gradient into water. Following blocking of endogenous peroxidase activity with 0.3% hydrogen peroxide for 10 min, the sections were washed with phosphate-buffered saline (PBS) and incubated overnight with rabbit anti-HMGB1 antibody (ab92310; Abcam; Cambridge, MA, USA) or HMGB2 antibody (ab11973; Abcam), VEGF antibody (ab1316; Abcam) or CD34 antibody (ab81289; Abcam) at the dilution of 1:100 in a humidified chamber at at 4°C. After washing with PBS, sections were incubated with biotinylated secondary antibody for 30 min at 37°C and then with horseradish peroxidase-labeled streptavidin for 30 min at 37°C. Diaminobenzidine (DAB) was used as chromogen and the sections were subsequently counterstained with hematoxylin, then dehydrated, cleared and mounted.

Sections were evaluated and scored as described previously by Yang *et al*(24); the extent of staining was scored as 0 (0%), 1 (1–25%), 2 (26–50%), 3 (51–75%) or 4 (76–100%), according to the percentage of positively stained areas. The staining intensity was scored as 0 (negative), 1 (weak), 2 (medium) or 3 (strong). The first and second scores were then added together to produce the final score (0–7) for HMGB1, HMGB2 or VEGF. Tumors with a final staining score of >3 were considered to have high protein expression and <2 were consi dered to have low protein expression.

CD34-stained whole sections were used to evaluate MVD as described previously by Ajili *et al* and Deniz *et al*(22,23). Microvessel counts of five areas with the most intense neovascularization were performed at a magnification of ×200.

### Statistical analysis

The χ^2^ test was used to analyze differences in categorical variables. Statistical analyses were performed using a Student’s two-tailed unpaired t-test for comparisons between two groups. All computations were performed with the Statistical Package for the Social Sciences (SPSS) 12.0 software (SPSS, Inc.; Chicago, IL, USA). P<0.05 was considered to indicate a statistically significant difference.

## Results

### HMGB1 and HMGB2 are overexpressed in BCa

**Figure 1:**
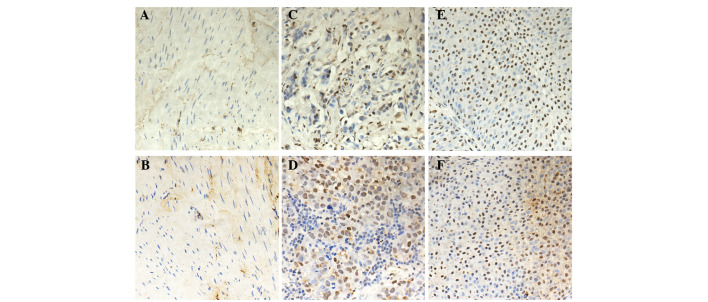
Immunohistochemical analysis of high mobility group box 1 (HMGB1) and HMGB2 expression in bladder carcinoma (BCa). HMGB1 (A) and HMGB2 (B) staining is negative in the majority of normal bladder urothelia. Low expression of HMGB1 (C) and HMGB2 (D) is evident in certain BCa. Extensive expression of HMGB1 (E) and HMGB2 (F) is shown in BCa. Original magnification, ×200.

Immunohistochemical analysis demonstrated that HMGB1 and HMGB2 were negative in the majority of normal bladder urothelia, but were positive in BCa (Fig. 1). A total of 35 (55%) cases and 19 (30%) cases had high expression of HMGB1 and HMGB2 protein, respectively (Table I). The correlation between HMGB1 and HMGB2 protein expression and the clinicopathological features of BCa demonstrated that levels of both HMGB1 and HMGB2 protein expression were significantly correlated with tumor grade and stage (Table I), and were not correlated with the remaining clinicopathological features tested, including gender and age.

### Upregulation of HMGB1 and HMGB2 mRNA in BCa

**Figure 2:**
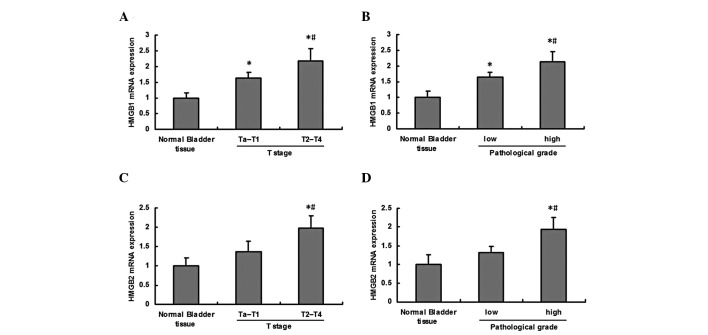
Real-time polymerase chain reaction (PCR) analysis of high mobility group box 1 (HMGB1) and HMGB2 mRNA expression in bladder carcinoma (BCa). (A) HMGB1 mRNA expression in BCa with different tumor (T) stages. (B) HMGB1 mRNA expression in BCa with different pathological grades. (C) HMGB2 mRNA expression in BCa with different T stages. (D) HMGB2 mRNA expression in BCa with different pathological grades. ^*^P<0.05 vs. normal bladder urothelium, ^#^P<0.05 vs. Ta-T1 stage or low pathological grade.

Real-time PCR analysis of HMGB1 mRNA expression revealed that the mRNA expression of HMGB1 was significantly upregul ated in BCa, and the mRNA expression in T2–T4 stage or high pathological grade tumor tissue was significantly higher than that in Ta-T1 stage or low pathological grade tumor tissue (Fig. 2A and B). The mRNA expression of HMGB2 was significantly upregulated in T2–T4 stage or high pathological grade tumor tissue compared with normal bladder tissue and Ta-T1 stage or low pathological grade tumor tissue; whereas no marked differences were observed between normal bladder tissue and low grade tumor tissue (Fig. 2C and D).

### Correlation between HMGB1 and HMGB2 protein expression and angiogenesis

**Figure 3:**
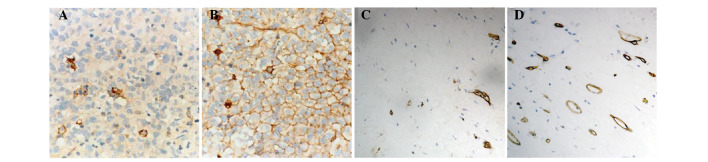
Immunohistochemical analysis of vascular endothelial growth factor (VEGF) and CD34 expression in bladder carcinoma (BCa). Low (A) and high (B) expression of VEGF in BCa. Low (C) and high (D) expression of CD34 in BCa. Original magnification, ×200.

To investigate angiogenesis in BCa, immunohistochemistry of VEGF and CD34 was performed. As demonstrated in Fig. 3 and Table II, levels of VEGF protein expression and MVD were significantly correlated with levels of HMGB1 and HMGB2 protein expression.

## Discussion

Our study found that HMGB1 and HMGB2 were overexpressed in BCa tissues compared with normal tissues, and were correlated with both the clinical stage and pathological grade of the tumor. In addition, overexpression of HMGB1/HMGB2 was also correlated with VEGF expression and MVD counts. This implies that HMGB1 and HMGB2 are likely to have key roles in the progression of BCa.

Overexpression of HMGB1 has been observed in several human tumor types. HMGB1 may be involved in cancer progression and prognosis, including apoptosis, angiogenesis, the inflammatory microenvironment, mobility, invasion, metastasis and patient survival (9,20). During cancer progression, HMGB1 has been observed to modulate the NF-κB, PI3K/AKT and mitogen-activated protein kinase (MAPK) signaling pathways by interacting with the receptor for advanced glycation end products (RAGE), while blockade of the HMGB1/RAGE interaction has been demonstrated to suppress tumor growth and metastasis (25,26). HMGB2 is highly homologous to HMGB1 and may exhibit similar effects in neoplastic development (27). Although HMGB1 has been studied more extensively, considerably less is known regarding HMGB2 in the study of cancer, particularly its relevance in carcinogenesis. To our knowledge, overexpression of HMGB2 has been identified in several types of tumors, including hepatocellular carcinoma (HCC) and skin cancer, and has been involved in cancer progression and prognosis via interacting with RAGE, steroid receptors, p53 and p73 (14,15). In addition, HMGB2 has been demonstrated to be significantly downregulated by the anti-human epidermal growth factor receptor 2 antibody through the AKT pathway in breast cancer cell lines (28). The present study indicated that HMGB1 and HMGB2 genes and proteins were overexpressed in BCa, using real-time PCR and immunohistochemical examin ation. The overexpression of HMGB/HMGB2 was correlated with both the clinical stage and pathological grade of the tumor, and HMGB1 and HMGB2 may be involved in BCa development and progression. Their involvement in the regulation of the carcinogenesis of BCa requires further investigation.

HMGB1 is a latent pro-angiogenic factor that has been observed to act both directly and indirectly, and an antibody targeting HMGB1 has been demonstrated to inhibit the angio-genesis *in vitro* and *in vivo*(29). HMGB1 was able to directly induce sprouting of endothelial cell spheroids in a collagen gel, and to stimulate endothelial cell proliferation, chemotaxis and repair of a wounded monolayer (30). HMGB1 exerts its angio-genetic effects via the RAGE pathway in tumor cells; it has been demonstrated to increase the expression of angiogenic growth factors, including VEGF, and to attract macrophages, which also produce a number of potent angiogenetic cytokines and growth factors (31). Furthermore, HMGB1 has been observed to attract endothelial progenitor cells (EPCs) and hematopoietic stem cells to sites of tissue injury and tumors to improve neovascularization mediated by RAGE, and anti-RAGE antibodies were able to inhibit this process (32). The role of HMGB2 in angiogenesis has not yet been studied. We found that overexpression of HMGB1 and HMGB2 were connected with VEGF expression and MVD counts in BCa tissues, indicating that they have important roles in angiogenesis, partly through VEGF. However, the precise molecular mechanisms require further study.

In conclusion, our data revealed that the expression levels of HMGB1 and HMGB2 were highly increased in BCa, and that HMGB1/HMGB2 overexpression was significantly correlated with malignant tumor progression and angiogenesis. As potential diagnostic and therapeutic targets, further studies are required to understand the molecular mechanism of the involvement of HMGB1 and HMGB2 in BCa.

## Figures and Tables

**Table I:** Correlation between HMGB1 or HMGB2 protein expression and clinicopathological features of the 64 patients with BCa.

Parameter	Total	High HMGB1 expression	P-value	High HMGB2 expression	P-value
Gender			0.637		0.154
Female	18	9		3	
Male	46	26		16	
Age (years)			0.451		0.784
≤65	32	16		10	
>65	32	19		9	
T stage			0.002		0.010
Ta-T1	42	17		8	
T2–T4	22	18		11	
Pathological grade			<0.001		0.016
Low	35	12		6	
High	29	23		13	

H]MGB, high mobility group box; BCa, bladder carcinoma.

**Table II:** Correlation between HMGB1 or HMGB2 protein expression and VEGF expression or MVD.

Parameter	Total	Low VEGF expression	High VEGF expression	P-value	MVD	P-value
HMGB1				<0.001		<0.001
Low expression	29	19	10		23.0±6.4	
High expression	35	7	28		35.5±10.8	
HMGB2				0.038		<0.001
Low expression	45	22	23		26.6±9.6	
High expression	19	4	15		37.6±10.3	

HMGB, high mobility group box; VEGF, vascular endothelial growth factor; MVD, microvessel density.
